# Supplementary material for: The HIV 5′ Gag Region Displays a Specific Nucleotide Bias Regulating Viral Splicing and Infectivity
Source: Viruses. 2021 May 27;13(6):997. doi: 10.3390/v13060997 (PMC8227319; doi:10.3390/v13060997)
Supplement: Supplementary file 1 [file viruses-13-00997-s001.zip › viruses-1222920-supplementary.pdf]

Supplementary Figure S1

```
                         MA                                      ISE
Gag wt      1    AGGAGAGAGATGGGTGCGAGAGCGTCGGTATTAAGCGGGGGAGAATTAGATAAATGGGAAAAAATTCGGTTAAGGCCAGG  80
                 |||||||||||||| || ||||| || ||  |    || || ||  | || || ||||| || ||  |  |  |||| ||
HuGag       1    AGGAGAGAGATGGGAGCCAGAGCCTCTGTGCTGTCTGGCGGCGAGCTGGACAAGTGGGAGAAGATCAGACTGCGGCCTGG  80

                           GFP
ΔMA-huGFP   1    AAGGAGAGAGATGGTGAGCAAGGGCGAGGAGCTGTTCACCGGGGTGGTGCCCATCCTGGTCGAGCTGGACGGCGACGTAA  80
                 ||||||||||||||| ||||| || || ||  | || || || || || || ||  | || ||  | ||||| |||||||
ΔMA-hivGFP  1    AAGGAGAGAGATGGTAAGCAAAGGAGAAGAATTATTTACAGGAGTAGTACCAATATTAGTAGAATTAGACGGTGACGTAA  80
```

The upper alignment represents wild type gag starting -9 nucleotides upstream of the matrix ATG (arrow) and its humanized counterpart. The previously described intron splicing enhancer (ISE) is depicted in light blue. The lower alignment compares the GFP sequence with its HIV-like derivative. Note, that the sequence upstream of the ATG is identical to wt HIV. Downstream of the ATG (arrow), the GFP ORF is located.

Supplementary Figure S2

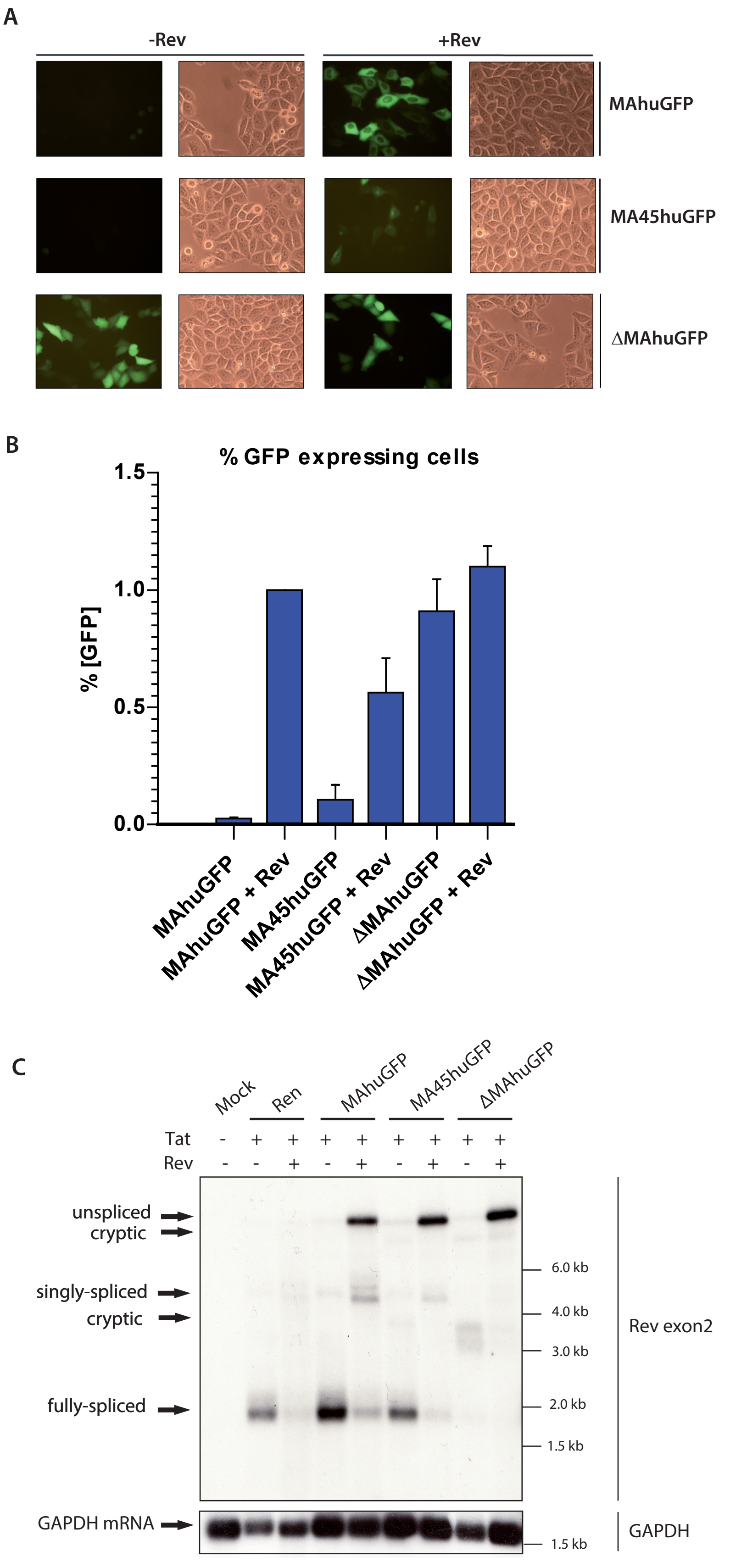

The huGFP insertion also results in Rev-independent expression in HeLa cells
(A) Light and fluorescence microscopy pictures of HeLa cells transfected with the different proviral constructs with or without a Rev expression plasmid. (B) Flow cytometry analysis of relative GFP expression levels of HeLa cells transfected with the indicated constructs. The percentage of GFP expressing cells was set to 1 for the MAhuGFP construct +Rev. (n=3). Error bars represent SD. (C) Northern blot of total RNA from HeLa cells transfected with the indicated constructs. The membrane was re-hybridized with GAPDH as a loading control. The different splice variant classes are indicated on the left, sizes are given on the right.
